# Operative Dentistry

**Published:** 1882-05

**Authors:** William Dutch


### OPERATIVE DENTISTRY.

BY WILLIAM DUTCH, D.D.S.

The principles underlying the mechanical part of the practice of dentistry, as it is daily presented to us, do not differ materially from those conceived by the founders of the profession. The same objects, viz., the salvation of teeth, which is striven for now called forth energetic efforts then.

Such rapid strides have been made in the adoption of methods (many of which have been discarded), so many new appliances have been brought into use, that, as we stand to-day, we can but wonder that teeth were filled at all fifty years ago.

So rapid, indeed, have been the changes, that a decade would mark the existence of one or more epochs in manipulative dentistry. New materials, and new methods of practice have been so numerous, following each other in such quick succession, that oftentimes good and tried things have had to fall back in order that new and perhaps inferior ones might take their places in the front rank of experimental dentistry.

Without wishing to be understood as placing any impediment in the way of still further investigation, the object of this paper is to counsel a momentary pause in the lightning-like march into new fields, and take a close survey of what we now have, or perhaps have neglected.

Different men, or men in different periods of professional life, will make use of materials and methods unthought of by others, and it is just such meetings as we are celebrating to-day which demonstrate the fact; and through this medium good ideas may be disseminated, interchanged, and absorbed. I might say that it is only through this medium that the little things in practice are taught. And if we would keep pace with the times it behooves us individually to take hold and help to "spread the light."

To begin. I desire to call attention to one of the past and almost discarded matters—I mean the use of gilded platina, or platina gold (it matters not what it may be called). Within the past year or two it has been my practice to give this preparation some consideration, and it now occupies an established place in my operating outfit. In certain kinds of teeth, and for some purposes, it is exceedingly useful. When thoroughly condensed and finished it presents a harder surface than gold alone. It may not be endowed with the same degree of cohesiveness, but it certainly stands in the mouth, even when built up on the articulating surfaces of teeth, better than gold. Again: the color, when polished, renders it less objectionable, if used in or upon dark blue teeth; and if properly managed in conjunction with pure gold, it admits of gradation in color.

As there is a growing desire on the part of a great mass of people—and some of the profession—to obtain some way by which dental operations may be performed with little labor and corresponding fees, I wish to speak of one way this may be accomplished in some cases. We sometimes meet with molar teeth containing large crown and buccal cavities, which, in consequence of the absence of antagonizing teeth. are non-masticators, or partially so at least. These cavities may be prepared just as would be done for the reception of any kind of filling, except that the margins present a regular, even form, filled nearly full with gutta-percha or wax, leaving the margins of the cavities clearly exposed. Impressions may be taken of the teeth, a model made of plaster, and pieces of pure gold plate, say twenty-two or twenty-four, Stub's gauge, nicely fitted to conform to the margins and the shape of the natural crown of a tooth. The inner surfaces of the gold pieces may be nicked with a sharp graver, presenting a roughened, retaining form. From the foregoing description it will be seen that these pieces of gold plate are only to occupy the surface of the cavities. The filling may be made of gutta-percha or any of the cements. If the latter be used, it will only be necessary to place the plate of gold in position, while the material is still in a plastic condition. If gutta-percha be used, the gold should be heated and carefully pressed to place, and the edges burnished down flush with the margins of the cavities. Durable operations may be made in this manner, saving the trouble and hard labor which attaches to packing in large gold fillings. I am not bringing this matter up, claiming any originality. For operations of this kind were frequently made years ago. I now allude to it simply to recall it to the minds of some who may not think of it.

It has been my experience of late years to see many fillings fail because of careless and faulty use of the file after filling. It seems to be the practice of some operators to pass the separating file between teeth to be treated on their proximate surfaces. After filling, the file is again passed through, trimming off the gold flush with the tooth, thereby leaving two parallel surfaces—or nearly so. We may almost always expect that space between teeth treated in this manner will in time become obliterated, when two flat surfaces will be brought in direct contact. It does not require a very astute mind to predict the result of this pernicious practice. Under such circumstances it is next to impossible to have these places kept clean without the use of the floss-silk, and it is only one in fifty persons who can be induced to use this means for cleanliness. The surface of teeth, after operating, should be left as nearly as possible as nature made them; that is, if any portion of the teeth must be in apposition, let it be the coronal portion, with sufficient space at or near the gum to admit of the introduction of the pick, and to allow the fluids of the mouth to flow through. If a little trouble to separate by pressure be taken, this can always be done. So thoughtless are some in this particular that it amounts almost to criminality; this action springing solely from the desire for gain.

Having met with so many cases of death of the dental pulp after filling, I cannot let this opportunity pass to utter a protest against the impunity with which some operators introduce large gold fillings in close proximity to the living pulp, even though it be not exposed. There is one fact in natural philosophy bearing upon this matter that must not be lost sight of, which is that the conductibility of a gold filling is always in direct ratio to its density. This may be regarded as explanatory of the fact that the old style of soft gold fillings would be, and were, tolerated by the pulp when placed in close proximity to it. I have met, in my own practice, cases wherein large, soft gold fillings have been removed and replaced by others made of cohesive gold, well impacted throughout; and although no portion of the dentine nearest the pulp had been removed, the pulps were found to have lost their vitality after a few months' time. I am well convinced that the cause was due to the increased amount of metal placed in a given space. So strongly am I impressed with the correctness of this course of reasoning, that I invariably interpose a capping of some sort over the floor of the cavity, especially in teeth of children.

As to the capping of exposed pulps, I have but little to say. My method is to place a piece of thin paper saturated with a solution of balsam of fir and chloroform over the exposure, even permitting it to extend well over the floor of the cavity, and form the capping of *os artificial*, or some kindred material. My object in using the paper is to avoid either mechanical or therapeutic irritation, which might be caused by the material applied.

Pivot teeth have been somewhat improved during the past year. Two or more forms have been introduced, and are kept in stock by our dental stores. Those of Dr. Bonwill seem most easily adjusted. In shape they are much like the ordinary pivot tooth, with the pivot chamber or hole passing entirely through, and are attached by means of a pin or screw, anchored with amalgam or cement extending into the root, and almost through the opening in the crown. When little strain or force is likely to be exerted, this method of inserting pivot teeth possesses advantages; but the appliance, when completed, seems to be lacking in strength, owing to the size of the chamber which receives the screw. The molars and bicuspids are especially deficient in this respect. There are other forms in the market, but it is questionable if any, when strength and durability are taken into account, are superior to the ordinary plate tooth, properly adjusted, for the six front teeth.

Some new appliances have come to light during the last year; more, however, in the way of improvements than any actually original inventions.

Johnson Bros.' new hand piece for the dental engine being the most important, perhaps, and is styled "Cone Journal Hand-piece, No. 2." The chief improvement lies in the fact that a cone-shaped enlargement is made on the shank of the burs, which is, by the locking device, drawn against the bearing at the end of the hand-piece. The principle on which this appliance is constructed certainly is good; and if it will stand the practical test, it will take a place second to none now in use. The expense of buying an entire new set of burs is overcome by a promise on the part of the manufacturers to alter old burs for use in the new hand-piece, at a nominal cost of 25 cents per doz. The locking device is not quite so simple as others now in use; but if a hand-piece has been given us that will run true and steady, under all circumstances, which this promises to do, that objection must yield to the increased advantages.

This hand-piece is so constructed that it may be attached to either the Morrison or White machines with equal facility.—*Trans. Cal. State Dental Association.*

